# Quercetin and Its Lecithin-Based Formulation: Potential Applications for Allergic Diseases Based on a Narrative Review

**DOI:** 10.3390/nu17091476

**Published:** 2025-04-27

**Authors:** Matteo Naso, Chiara Trincianti, Maria Angela Tosca, Giorgio Ciprandi

**Affiliations:** 1 Allergy Center, IRCCS Istituto Giannina Gaslini, 16100 Genoa, Italychiaratrincianti@gaslini.org (C.T.); mariangelatosca@gaslini.org (M.A.T.); 2 Allergy Clinic, Casa di Cura Villa Montallegro, 16100 Genoa, Italy

**Keywords:** quercetin, type 2 inflammation, antioxidant activity, allergic diseases, food supplements, phospholipids, phytosome

## Abstract

Quercetin, a natural flavonoid, present in various vegetables and fruits, has garnered increasing attraction for its potential antiallergic properties. Its broad-spectrum activity depends on its anti-inflammatory, immunomodulatory, and antioxidant effects, which target the critical pathways involved in type 2-driven allergic inflammation. Quercetin inhibits mast cell degranulation, reduces the production of histamine and pro-inflammatory cytokines, and restores homeostasis of the immune system by modulating the Th1/Th2 and Treg/Th17 balances. Additionally, its antioxidant properties help to dampen oxidative stress, a critical factor in the pathophysiology of allergic diseases. In vitro studies have consistently demonstrated quercetin’s ability to suppress allergic reactions. In contrast, in vivo studies, particularly in murine models of allergic rhinitis, have confirmed its efficacy in relieving symptoms (such as nasal itching, sneezing, rhinorrhea, and congestion) and dampening type 2 mucosal inflammation. Preclinical evidence also supports its therapeutic potential in asthma, conjunctivitis, atopic dermatitis, and food allergies. However, human studies are still scarce, as only two clinical trials investigated quercetin as a monotherapy. Both studies reported promising results, including symptom reduction and improved quality of life, though larger, randomized trials are needed to validate these findings. Some other studies have investigated multicomponent products that also contain quercetin. This review aimed to report and discuss the most recent in vitro and in vivo evidence on quercetin’s application in allergic models. It also provides a comprehensive overview of human studies, highlighting its potential as an agent in food supplements to manage patients with allergic diseases. Moreover, this review introduces a new quercetin phospholipids formulation that may represent a keystone in clinical use. The literature search was based on a PubMed consultation considering the most recent (last five years) publications using the keywords “quercetin and allergic disease” and “quercetin and immune system”.

## 1. Introduction

Quercetin is a flavonol, one of the six subclasses of flavonoid compounds, part of the more prominent family of polyphenols. The name is derived from *quercetum* (oak forest) and has been used since 1857. The International Union of Pure and Applied Chemistry (IUPAC) nomenclature for quercetin is 3,3′,4′,5,7-pentahydroxyflavanone (or its synonym 3,3′,4′,5,7-pentahydroxy-2-phenylchromen-4-one) [1].

Quercetin is one of the most abundant dietary flavonoids in fruits and vegetables. It can be found in various plants worldwide, including onion, caper, apple, berry, tea, tomato, grape, *Brassica* vegetables, shallot, nuts, seeds, barks, flowers, and leaves. 

Quercetin is also found in medicinal botanicals, including *Ginkgo biloba*, *Hypericum perforatum*, and *Sambucus Canadensis* [2]. The highest concentration of this polyphenol is found in capers, with a mean value of 172–234 mg/100 g, while the lowest is in black or green tea, with a mean value of 0.72–2.74 mg/100 mg [3,4,5].

As a widely distributed compound in various plant species, quercetin accounts for approximately 75% of the daily intake of flavonoids, with some variation depending on the dietary habits of the populations [1]. Quercetin is mainly found in nature in the form of glycosides, i.e., with different sugars that are bound to its hydroxyl groups [6].

**Figure 1:**
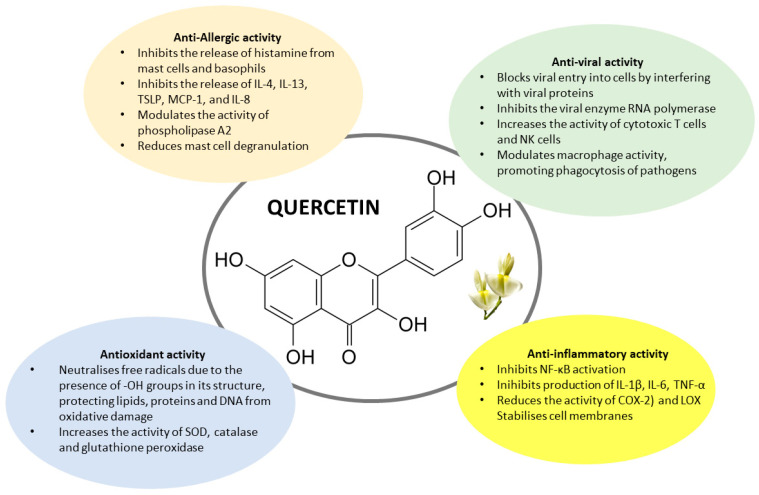
Chemical structure and mechanisms of action of quercetin. Quercetin exerts many mechanisms of action, including anti-allergic, anti-viral, antioxidant, and anti-inflammatory activities. For more details, see the text and Table 1.

Quercetin is an attractive molecule with several beneficial activities, including antiallergic, antioxidant, anti-inflammatory, and antiviral, as schematized in Figure 1 and Table 1. Therefore, the present review aimed to report and discuss the most relevant evidence and reasons for its potential use in allergic diseases.

## 2. Methods

This narrative review was performed following three steps: conducting the search, reviewing abstracts and full-texts, and discussing results. For this, the PubMed, database was searched to identify the relevant studies, according to the development of the review. The final search was conducted in February 2025 and included English language-based international articles, online reports, and electronic books. The keywords “quercetin and allergic disease” and “quercetin and immune system” were used. After the complete search, the abstracts were read to ensure that they addressed the topic of interest. All duplicates were removed, and the abstracts of the remaining articles were reviewed to ensure that they address the review inclusion criteria. The eligible criteria were studies that investigated quercetin in at least one of the three aspects (in vitro, animal, and human studies). Therefore, these studies of interest were summarized and synthesized to integrate the narrative review. Since it is a narrative review, it was unnecessary to document the literature search on specific platforms.

## 3. Pharmacokinetics of Quercetin

The absorption of quercetin varies depending on the sugar with which it is conjugated. According to the literature, quercetin glucosides (found in onion and shallot) are better absorbed than rutinosides. In addition, absorption is influenced by the matrix of the food in which it is contained and by the co-administration of fibers and fats. Quercetin is stable in gastric acid and is probably absorbed in the upper segment of the small intestine [21,22]. The estimated absorption of quercetin glucoside, the naturally occurring form of quercetin, ranges from 3% to 17% in healthy individuals receiving 100 mg orally [1]. Quercetin is more completely absorbed than quercetin in the aglycone form, and the simultaneous intake of vitamin C, folate, and additional flavonoids improves its bioavailability [23].

Regarding metabolism, quercetin undergoes glucuronidation, sulfation, and methylation processes in various organs, including the small intestine, colon, liver, and kidney. The small intestine and liver are the main sites of biotransformation. Continuous diet intake containing quercetin is related to increased plasma levels [24,25,26].

The available literature suggests that quercetin and its metabolites accumulate in the organs involved in its metabolism and excretion and appear to be distributed primarily within the mitochondria [27].

The main excretory organ of quercetin is the kidney. Excretion is prolonged as quercetin has a medium-long half-life of 11–28 h [28]. Another important drawback of quercetin is represented by its low solubility (about 0.01 mg/mL [29]) and low stability that impact with bioavailability, which is poor and not always dose-related [30]. In addition, several efforts have been made to improve solubility and bioavailability, as described in Section 7 (see below).

## 4. Quercetin and the Potential Effect on Allergic Disease

Allergic diseases share a type 2 inflammation, characterized by a type of immunity polarization [31]. Briefly, a functional and allergen-specific defect of T- and B-regulatory cells promotes the expansion of innate and acquired immunity toward type 2. As a result, type 2-dependent cytokines (IL-4, IL-5, and IL-13) induce, maintain, and amplify allergic inflammation, sustained by eosinophilic infiltration of target tissues [32]. As allergy is an inflammatory disease, anti-inflammatory, antioxidant, immunomodulatory, and anti-allergic compounds could dampen type 2 inflammation and relieve allergic symptoms [33].

Consequently, considering its mechanisms of action, quercetin might theoretically have a preventive and therapeutic add-on role in managing allergic diseases. However, the molecular and cellular mechanisms have yet to be precisely discovered. Among its known and widely studied properties, quercetin has strong antioxidant power. Quercetin can inhibit the initiation phase of chain oxidation and prevent chain propagation [34]. This effect is primarily due to its flavonoid structure, consisting of a 2,3 double bond in combination with a 4-oxo bond in the C ring, which is remarkably efficient at scavenging free radicals. In addition, the presence of 3-OH groups potently inhibits lipid oxidation [35,36].

This ability has also been studied in the model of allergic diseases. In fact, certain factors, including exposure to secondhand smoke and viruses, can lead to secondary ciliary dysfunction that increases the residence time of antigens and irritating agents on the mucosa of the airways. This stasis upregulates the oxidant enzymes promoting the production of O_2_ and, consequently, oxidative stress [37]. Using quercetin could reduce the oxidative stress that characterizes all types of inflammation.

Beyond its antioxidant properties, it has been hypothesized that quercetin may also have antiallergic properties due to its ability to influence several pathogenetic mechanisms involved in allergic diseases. Quercetin inhibitory activity directly affects mast cells, stabilizing them and reducing mediator release by the inhibition of the Ca^2+^ influx, histamine, leukotrienes, prostaglandins, and activation of protein kinase [38,39]. Fewtrell and Gomperts [40] offered the first evidence in 1977, when the authors observed quercetin‘s direct inhibitory effect on the release of histamine from mast cells, demonstrating its effect on Ca^2+^ flux. Several other studies have also demonstrated a strong reduction of intracellular Ca^2+^ mobilization in mast cells [41]. This inhibitory effect on mast cells lowers the release of other mediators, such as tryptase, monocyte chemoattractant protein 1 (MCP-1), and IL-6 [42].

Quercetin also exerts a significant inhibitory effect on interleukins (IL) that plays a crucial role in promoting allergic inflammation, i.e., suppressing the production of IL-4 and IL-13 by allergen- or anti-Immunoglobulin E (IgE) antibody-stimulated receptor-expressing cells [43]. Moreover, quercetin induces relevant genetic expression and production of T helper (Th) Th-1-derived interferon (IFN)-γ and inhibits enzymes such as lipoxygenase and cyclooxygenase, reducing the production of molecules like thromboxane, prostaglandins, and leukotrienes involved in type 2 reactions [44]. In addition, quercetin has demonstrated the ability to modulate Th type 1 and 2 cells (Th1/Th2) and inhibit allergen-specific IgE (Immunoglobulin E) antibody production [43,45].

There are few reviews [3,46] in the literature on quercetin and its potential effect on allergic disease, and all are focused mainly on in vitro and animal studies. To date, no reviews have summarized the available literature on the efficacy of quercetin in clinical trials. The aim of this review is to gather the most recent evidence available in the literature on the possible effects of quercetin in allergic disease in vitro and animal studies and to review all the evidence of quercetin and its effect on allergic diseases from human studies available in the literature.

## 5. Quercetin and Allergic Diseases: In Vitro Studies

Quercetin has demonstrated various anti-inflammatory, immunomodulatory, and antioxidant effects in various in vitro studies, highlighting its therapeutic potential in allergic and inflammatory diseases (Table 2).

Among the antioxidant effects, Edo et al. studied the impact of quercetin on Thioredoxin (TRX) production and TRX mRNA expression in human nasal epithelial cells (HNEpC) stimulated with H_2_O_2_. They found that cells treated with 1.0 nM quercetin increased the levels of H_2_O_2_-induced TRX. TRX is a key antioxidant protein that reduces oxidative stress by catalyzing disulfide bond reduction in proteins, protecting cells from damage, and regulating redox-sensitive signaling with a possible immunomodulatory effect [47].

Isoquercetin, a quercetin glycoside, was shown to significantly suppress the production of histamine and pro-inflammatory cytokines, such as IL-6, IL-8, IL-1β, and tumor necrosis factor (TNF)-α, in phorbol-12-myristate 13-acetate plus the calcium ionophore A23187 (PMACI)-stimulated human basophilic KU812 cells. This effect correlated with the suppression of extracellular signal-regulated kinase (ERK) phosphorylation, emphasizing the role of the ERK- Mitogen-activated protein kinase (MAPK) pathway in isoquercetin-mediated allergy inhibition [7].

**Figure 2:**
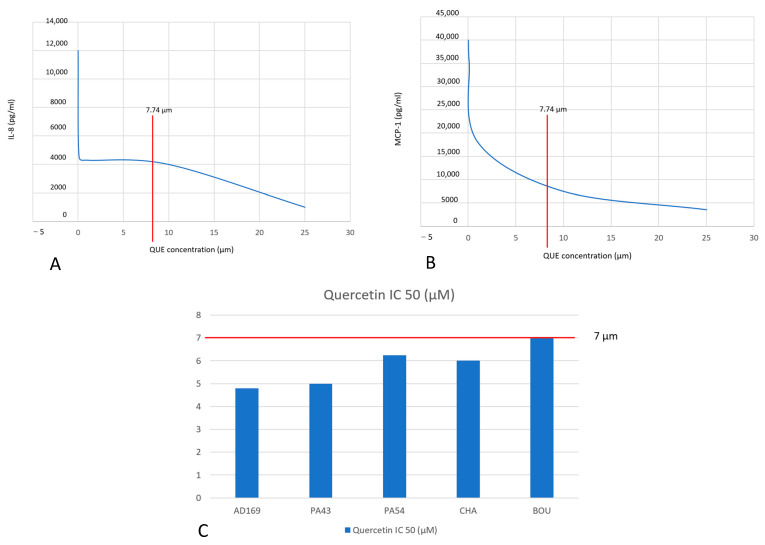
Quadrant (**A**): Effect of quercetin on human bronchial epithelial cell IL-8 expression induced by TNF-α. Partly modified from Ref. [10]. Quadrant (**B**): Effect of quercetin on human bronchial epithelial cell MCP-1 expression induced by TNF-α. Partly modified from Ref. [10]. Quadrant (**C**): Antiviral effect of quercetin on HCMV reference strain AD169 and four clinical isolates (two naïve isolates from newborns, one ganciclovir-resistant, BOU, and one multi-drug resistant, CHA). Partly modified from Ref. [11].

Quercetin exhibits remarkable inhibitory effects on mast cell degranulation and related calcium signaling. In the “laboratory of allergic diseases 2” (LAD2) human mast cell line, quercetin reduced Substance P- and C48/80 (a compound used to promote histamine release from mast cells in experiments)-induced calcium flux in a dose-dependent manner, with concentrations above 100 μM demonstrating effects comparable to dexamethasone at 100 μM. Additionally, quercetin attenuated the release of β-hexosaminidase, histamine, monocyte chemoattractant protein-1 (MCP-1), and IL-8 [9]. Similarly, in this study, where LAD2 cells were incubated with IgE, quercetin inhibited DNP-HSA/IgE-induced Ca^2+^ flux, significantly suppressed the DNP-HSA/IgE-induced release of β-hexosaminidase and histamine, and inhibited the DNP-HAS/IgE-induced secretion of IL-13, MCP-1, IL-8, and TNF-α in a concentration-dependent manner (0, 25, 50, 100 μM), as reported in Figure 2, quadrant A and B [10].

Quercetin also acts on periostin, a potential biomarker of type 2 inflammation. The authors investigated the influence of quercetin on the production of both periostin and periostin-induced eosinophil chemoattractants from HNEpC stimulated with IL-4 in vitro. Quercetin suppressed periostin production by HNEp cells stimulated with IL-4. Quercetin also reduced periostin-induced eosinophil chemoattractants, such as those regulated on activation, normal T-cell expressed and secreted “regulated upon activation, normal T-cell expressed and secreted” (RANTES), and eotaxin from HNEpC [48]. In addition, quercetin was found to suppress nitric oxide (NO) production from HNEp IL-4 stimulated cells. NO plays a dual role in type 2 inflammation. It is a signaling molecule that can exacerbate inflammation by promoting vasodilation, immune cell recruitment, and cytokine production. However, excessive NO, often produced by inducible nitric oxide synthase (iNOS), amplifies Th2-driven responses, contributing to airway hyperresponsiveness and tissue damage in allergic inflammation [49]. Quercetin may also affect asthma: in a study, the flavonol could attenuate acetylcholine chloride (ACH) and K^+^ inducted contraction in mouse and human airway smooth muscle [50]. Additionally, quercetin enhanced the production of Clara cell 10-kD protein (CC10), suppressing the action of phospholipase A2 and type 2 machinery. Otaki et al. showed that HNEpC stimulated with TNF-α increased CC10 production when treated with quercetin at a dosage of at least >5.0 µM [51].

Regarding the immunomodulatory role of quercetin, Tanaka et al. studied the effect of quercetin on human peripheral-blood CD^4+^ T cells cultured with IL-4, measuring IL-5, IL-13, and INF-γ. Quercetin at more than 5.0 µM inhibited the synthesis of IL-5 and IL-13 from CD^4+^ T cells. On the other hand, quercetin at concentrations higher than 5.0 µM suppressed the negative action of IL-4 on INF-γ production from CD^4+^ T cells [52].

Lastly, quercetin also showed activity in an atopic dermatitis model of human keratinocyte HaCaT cells incubated with IFN-γ/TNF-α, demonstrating decreased inflammation with the suppression of the production of IL-1α, IL-1β, IL-6, IL-15, monocyte chemoattractant protein (MCP)-1, MCP-3, chemokine ligand (CCL)17 and CCL22, and protection from oxidative stress and apoptosis [8]. The same results were observed in another study, where HaCat cells were incubated with IL-4, -13, and TNF-α in vitro and treated with quercetin. Pre-treatment of the cells with 1.5 mM of quercetin significantly reduced the expression of AD-induced IL-1b, IL-6, IL-8, and thymic stromal lymphopoietin (TSLP), while it strongly enhanced the expression of superoxide dismutase-1 (SOD1), SOD2, catalase, glutathione peroxidase, and IL-10 [53].

However, in vitro studies have some limitations, mainly concerning the low solubility, stability, and bioavailability of quercetin. As a result, it is very difficult to compare in vitro/in vivo effective concentrations, as deeply discussed in Section 3 and Section 7.

## 6. Quercetin and Allergic Diseases: Animal Studies

Quercetin has demonstrated potent anti-inflammatory, antioxidant, and immunomodulatory effects in various preclinical (animal) models of allergic and inflammatory diseases (Table 3).

In C57BL/6 mice with C48/80-, or Substance P-induced paw edema, quercetin was administered at different dosages (0, 1.0, 2.0, and 4.0 mg/mL), and a reduction of paw edema thickness, and vasodilation was observed in a dose-dependent manner. Furthermore, mice serum histamine, MCP-1, and IL-8 release were inhibited in response to quercetin administration, and the degranulation induced by C48/80 or Substance P was inhibited through reduced C48/80 induced calcium flux in peritoneal mast cells in a dose-dependent manner (0, 50, 100 and 200 μM). Moreover, quercetin attenuated the release of histamine and MCP-1 in peritoneal mast cells, suggesting an anti-pseudo-allergic effect of quercetin [9].

Animal models of allergic rhinitis have been extensively used to demonstrate the effectiveness of quercetin in reducing both symptoms and releasing type 2 inflammation mediators. In the ovalbumin (OVA)-induced allergic conjunctivitis C57BL/6 mice model, quercetin was able to reduce IgE, Histamine, IL-4, and TNF-α, substance P in a concentration-dependent manner in the AC mouse model, and the effect was similar to mice treated with olopatadine [10]. Edo et al. studied the effects of quercetin on the AR mouse model in OVA-sensitized BALB/c mice. The mice were orally administered quercetin at 10.0–25.0 mg/kg daily for five consecutive days. Quercetin significantly reduced lipid peroxide levels at nasal level, while the dosages of 20.0 and 25.0 mg/kg of quercetin, but not 10.0 and 15.0 mg/kg, increased nasal TRX levels [47].

Similarly, in a study [54], where mice were sensitized to toluene 2.4-diisocyanate (TDI), replicating a model of AR, oral administration of quercetin for 5 and 7 days, but not 2 and 3 days, could inhibit sneezing and nasal rubbing movements, which were increased by TDI nasal challenge. The minimum dose that caused significant inhibition was 25 mg/kg. Furthermore, an oral administration at more than 25 mg/kg for 5 consecutive days significantly inhibited the increase in SP, calcitonin gene-related peptide (CGRP), and nerve growth factor (NGF) contents in nasal lavage fluids induced by TDI nasal challenge. In addition, Otaki et al. also demonstrated that the oral administration of at least 25mg/kg quercetin in TDI-sensitized rats reduced TDI-induced allergic symptoms. Moreover, it was demonstrated that administering oral quercetin at the same dosage could affect the nasal production of CC10 following antigenic stimulation [51].

Mu et al. [55] demonstrated that nasal application of quercetin decreased nasal symptoms and mucosal levels of IgE, IL-17, TNF-α, and IL-6 and improved the damage caused to the mucosa by allergic inflammation in an AR mouse model.

Sagit et al. [56] demonstrated that quercetin could reduce OVA-specific IgE in a mouse model of AR by topical application of nasal steroids. This result was confirmed both by microscopic findings, in which vessel dilation, mucosal inflammation, and dilation of serous gland ducts were markedly reduced in mice treated with quercetin or topical nasal steroids compared to the control group, and by histopathological data that showed increased expression of Cyclooxygenase (COX)-2 and vasoactive intestinal polypeptide (VIP) in the control group. In a similar murine model of AR, in addition to decreasing symptoms after nasal challenge, quercetin was able to reduce Th17 cells, increase Treg cells, as well as promote Th1/Th2 balance, by reducing IgE and IgG1 levels and elevating IgG2 levels and the IgG2a/IgG1 ratio in a dose-dependent manner. Quercetin also decreased the serum levels of histamine IL-4 and IL-5 and downregulated the levels of IL-17 and TGF-ß in nasal lavage fluid. Moreover, quercetin inhibited OVA-induced activation of the NF-κB pathway [57]. A similar result about the Th1/Th2 and Th3/Th17 ratio was found in this animal model of food allergy, where the administration of quercetin conjugated to the allergen decreased its induced damage to the intestinal barrier [58].

About atopic dermatitis, in a mice model, quercetin was able to suppress MC903 (a low-calcemic analogue calcipotriol of vitamin D3)-induced AD skin lesions. Furthermore, quercetin suppressed inflammatory cytokines, such as IFN-γ, CCL17, CCL22, TNF-α, IL-4, and IL-6, expression in lesions of MC903-induced AD-like mice [8]. Quercetin has shown activity in animal models of allergic airway inflammation; in this study, mice with OVA-induced asthma were treated with quercetin intraperitoneally, and its efficacy was compared to that of dexamethasone treatment. Both treatments decreased the thickness of the epithelium and subepithelial smooth muscle, and the number of mast cells and goblet cells was inferior in the lung tissues of mice treated with quercetin and dexamethasone. These data were also confirmed by the lower immunoscoring of IL-25, IL-33, TSLP, TUNEL, and caspase-3 compared to untreated mice with allergic airway inflammation. Finally, in bronchoalveolar lavage (BAL), quercetin and dexamethasone resulted in lower levels of IL-4, IL-25, IL-33, and TSLP in BAL and OVA-specific IgE in serum compared to levels in untreated mice with the allergic airway inflammation group [59].

Effects on oxidative stress, tissue remodeling, and inflammation were also observed in a more recent study where quercetin was capable of reducing GATA binding protein 3, TNF-α, transforming growth factor (TGF)-b1, IL-1b, and Acta2 gene expression. In addition, quercetin augmented the Tbx21 expression in asthma model. Moreover, quercetin also reduced oxidative stress by diminishing malondialdehyde (MDA) levels and increasing total antioxidant capacity (TAC), catalase (CAT), SOD, and glutathione peroxidase (GPX) levels. Furthermore, quercetin decreased IL6 and TNFα levels and increased IL10 levels in lung tissue after treating asthma with quercetin [20].

However, similarly to in vitro studies, animal studies also have some limitations, as the used models display some methodological biases, and the animal model is not directly superimposable to the human model. For example, allergen exposure in animal models is completely different from natural allergen exposure and the immune response of allergic patients is far different from experimental animal studies. On the other hand, animal studies make it possible to perform experimental approaches not easily executable in humans.

## 7. In Vivo Studies: Multicomponent Nutraceuticals (Containing Quercetin Among Ingredients) Studies on Humans

There are many nutraceutical products containing quercetin on the market. However, only some studies are available in the literature, and most of them were conducted with products containing several active substances, including quercetin (Table 4).

In 2002, Hu et al. studied the efficacy in patients with allergic rhinitis of a compound of 11 traditional Chinese herbs, containing 460 mg of *Ginkgo biloba (GB)*, containing quercetin [60]. So, 58 patients randomly received capsules of the compound or placebo in doses of five capsules twice daily for 12 weeks. Results showed a statistically significant improvement in some of the symptoms of AR, whereas others exhibited a positive trend that did not reach statistical significance.

Moreover, one year after completion of the 12-week treatment, the active group had an average improvement of VAS scores (including less rescue medication) of 27%. In comparison, the average improvement of the VAS scores for the placebo group was only 3%. Although encouraging, this experience showed the effects of a compound of 11 traditional Chinese herbs on patients with AR. Unfortunately, it was impossible to quantify the direction of quercetin [60].

As mentioned before, quercetin is found in onions and shallots. In this randomized, double-blind, placebo-controlled trial, patients with AR were administered with shallot extract and cetirizine or cetirizine alone for 4 weeks; 62.5% of patients in the shallot group and 37.5% of patients in the control group showed improvement of post-treatment VAS. Moreover, ocular symptoms improved only in patients treated with shallot, as confirmed by a significant variation in the total ocular symptom score (TOSS) [61].

The first experience available in the literature on an oral food supplement containing quercetin, and not contained within a plant ingredient of the compound, dates back to 2015, when Ariano conducted an open-label study with an oral supplement containing dry extract of *Perilla frutescens* 80 mg (containing several flavonoids), quercetin 150 mg, and vitamin D3 5 mcg (200 IU). This author enrolled 23 adult patients with perennial AR in treatment with standard antiallergic drugs and evaluated symptoms at baseline and after 4 weeks of treatment with the compound. The results were encouraging, showing a highly significant reduction of the overall symptoms: approximately 70% for symptom scores and 73% in the use of antiallergic drugs. Moreover, all allergic symptoms, such as sneezing, rhinorrhea, nasal obstruction, ocular itching, lacrimation, and congestion of the conjunctiva, showed a significant reduction [62]. The efficacy of the same compound was also observed in pediatric patients. In 2019, Marseglia et al. conducted a randomized, double-blind, placebo-controlled (RDBPC) trial in 146 polysensitized AR pediatric patients randomly stratified into two groups, treated for 4 weeks with compound plus standard treatment or placebo plus standard treatment [63]. Both groups significantly (*p* < 0.0001 for both) reduced Total Symptom Score (TSS) after 4 weeks (% change: −63.6% in compound-group and −60.7% in placebo-group; *p* = not significant in intergroup analysis). Notably, 24 children had symptoms worsening between week 2 and week 4: 8 in the compound group and 16 in the placebo group, with significant intergroup differences (*p* < 0.05). The study mentioned above included a phase II study [64] that was an open-label, parallel-group, extension study in which patients treated with the study product in Period I continued treatment with the nutraceutical supplement.

In contrast, patients initially treated with a placebo received no further treatment. Of the 146 patients previously enrolled, 128 continued the study and were divided into two arms, of which 64 were assigned to open-label oral food supplement treatment (OFST group) and 64 to observation only (Observation Group: OG). The OFST group showed a significant difference in the duration of symptom-free days compared to the OG (Log-Rank test = 4.16; *p* = 0.0413), with a Hazard Ratio (HR) 0.54 (CI 95% 0.29–0.99), and there was a significant difference in AR exacerbations, where only 25% of the OFST group had an AR exacerbation compared to 42% of the OG (*p* = 0.039). Analyzing exacerbations, patients in the LG group needed fewer days of rescue medication than the OG group (*p* = 0.0018). In addition, those children who were taking other medications together with the compound had fewer AR flare-ups than those taking only medications (*p* = 0.051). This phase II study proved that this food supplement effectively guaranteed a long-lasting symptom-free period. At the end of these two studies, some patients from both groups were observed for one year. During this phase, patients could take antihistamines on demand, and their use was reported in a diary. The patients in the OFST group had a lower median number of days of antihistamine therapy compared to OG, and the difference was statistically significant (*p* = 0.008) [65]. In addition, this compound has shown activity in the allergic inflammation of the airways in patients with AR. It is well known that small airways are early involved in AR. A pathological value (<65% of predicted) of the forced expiratory flow between 25% and 75% of the vital capacity (FEF_25–75_), could suggest subclinical asthma [71]. After one year of observation, the OFST group had significantly higher MEF_50_ than OG children (*p* = 0.009) [66]. Another retrospective study was performed on the population of the studies mentioned above to evaluate the number of respiratory infections (RI) and the use of antibiotics. So, 53 patients with AR were retrospectively enrolled from the OFST and OG groups, and patients from the OFST group had a significant reduction in the number of recurrent infections and antibiotic course compared with OG (*p* = 0.01 and 0.002, respectively) [67]. This effect might also depend on the antiviral activity exerted by quercetin, as reported in Figure 2, quadrant C [11].

In the studies available in the literature regarding topical effects of quercetin in the organs affected by allergic diseases, it was only present as a component of an herb or compound. In the RDBPC study, a GB and hyaluronic acid (HA) eyedrop compound was utilized to treat seasonal allergic conjunctivitis, and its effects were compared with hyaluronic acid treatment. Patients treated with GB–HA had a significant improvement of ocular symptoms. GB contains several active substances such as quercetin, ginkgetin; kaempferol; isorhamnetin; procyanidin; prodelphinidin; Ginkgolids A, B, C, J, and M; and bilobalides. This study emphasized the potential role of GB, and especially quercetin, in seasonal allergic conjunctivitis, but even in this case, it was not possible to directly assess the effects of quercetin [68].

Topical effects of compounds containing quercetin were also observed in a proof-of-concept study where 12 adult patients affected by AR were treated with the nasal application of extract of *Artemisia abrotanum* L. Patients experienced a relevant relief of nasal and ocular symptom scores (*p* < 0.01 and <0.05, respectively) [69].

Moreover, Wang et al. treated asthmatic children with a combination of aerosolized *A. membranaceus*, budesonide, and terbutaline or with aerosolized budesonide and terbutaline alone. The group treated with *A. membranaceus* had better improvement in lung function than the control group (*p* < 0.05), and *A. membranaceus* showed higher efficacy in preventing the occurrence of childhood (*p* < 0.05). In addition, the authors observed that the serum levels of IgE, IL-17, and IL-23 were reduced significantly in the treatment group when compared with the control group, while the serum levels of FoxP3 and TGF-β were increased in the treatment group when compared with the control group (*p* < 0.05). Finally, patients treated with *A. membranaceus* showed an increased percentage of Treg cells and a reduced percentage of Th17 cells, showing the capacity to modulate the balance of Treg/Th17 cells [70].

Globally considering these studies using multicomponent compounds, a relevant limitation has to be underlined. It is very difficult to extrapolate the real direct effect of each component, and a synergic activity could occur. Consequently, these studies cannot be considered direct proof of a specific effect exerted by quercetin alone.

## 8. In Vivo Studies: Single-Component Nutraceuticals (Containing Only Quercetin) Studies on Humans: A New Formulation

Very few studies are concerned with using quercetin-only for benefits on human allergic discomfort (Table 5).

The clinical studies reported in the literature are mostly on a new formulation of quercetin dispersed in phospholipids [73,74,75,76].

Bioavailability represents a drawback for quercetin, so a new formulation has been developed to ameliorate the absorption rate. The new formulation of quercetin, called Quercetin Phytosome™ or Quercefi™, is an innovative delivery form of quercetin formulated with a specific food grade technology to optimize its bio-absorption and performance. This novel phospholipids-based delivery system is standardized to contain ≥34.0% ≤42.0% of quercetin by HPLC [75].

Phytosome™ technology, differently from complexes and liposomes, is a solid dispersion of botanicals or natural compounds into a food-grade matrix based on lecithin (phospholipids), amphipathic molecules which act as inhibitor of self-aggregation and effective wetting agent. This innovative approach boosts the efficacy and targeting of various natural compounds, optimizing their interaction with the microbiome, enhancing their bio-absorption, and improving their stability and tolerability [77].

This lecithin-based formulation offers numerous advantages: it is made by food-grade ingredients, so it is safe and well tolerated over time; it does not contain nanoparticles or synthetic adjuvants; and it can be tailored and optimized for several phytonutrients. Therefore, in addition to quercetin, Phytosome technology was applied to other natural ingredients, including *Berberis aristata* extract, Coenzyme Q10, *Melissa officinalis* extract, and Curcumin [78,79,80,81]. Furthermore, Phytosome ingredients could be applicable for various dosage forms from traditional capsule and tablets to orodispersable powder, shots, gums, and gummies.

**Figure 3:**
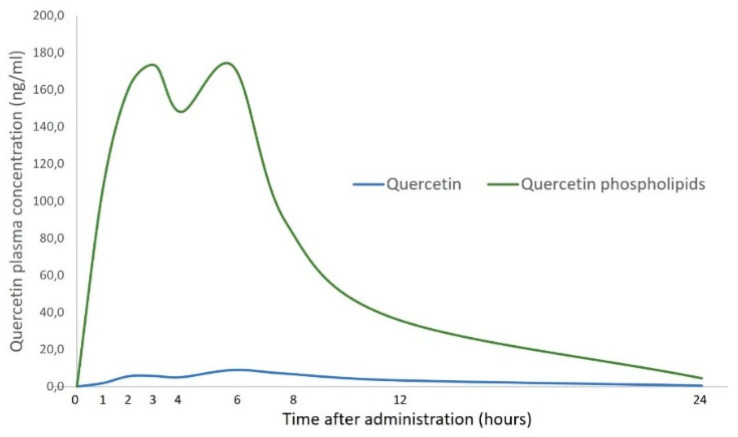
Plasma concentration of quercetin and its lecithin-based formulation. This pharmacokinetics study compared two groups of healthy volunteers supplemented with quercetin or quercetin phospholipids. Details are reported in the text. Partly modified from Ref. [73].

This novel formulation of quercetin was initially developed and tested to evaluate its potential advantages in terms of both solubility and bioavailability [73]. In vitro studies conducted in simulated gastric and intestinal fluids revealed that quercetin phospholipids achieved a remarkable improvement in solubility—up to 11 times greater—compared to the unformulated compound. The pharmacokinetic profiles of both quercetin and its lecithin-based formulation were evaluated in 12 healthy human volunteers. This study demonstrated that quercetin phospholipids has significantly ameliorated both in vitro solubility and oral absorption (concerning exposure and maximum concentration achieved) compared with the unformulated quercetin, as reported in Figure 3.

Another in vitro model evaluated the human microbial metabolism of quercetin derived from unformulated and quercetin phospholipids [77]. These substances were initially identified for their profile in native (poly)phenols and then fermented with human fecal microbiota for 24 h. After 24 h, the native quercetin concentrations in both compounds were significantly reduced (*p* < 0.05) upon interaction with fecal microbiota, with reductions of 93% and 50%, respectively, for the unformulated and lecithin-formulated products, respectively. Consequently, the degradation of quercetin was faster in the unformulated product, indicating lower in vitro stability of native quercetin in the unformulated version compared to the lecithin-formulated one. In that study, new perspectives were investigated on the role of phospholipids as delivery systems on influencing the microbial metabolism of quercetin’s flavonols in the colonic environment, underlying the role of the formulation to optimize bioactivity associated to their intake.

Regarding the health benefits on allergy, successively, Cesarone et al. [74] enrolled 58 patients with allergic asthma (AA) and allergic rhinitis; 30 subjects were managed with quercetin phospholipids (20 with 200 mg per day, and 10 with 100 mg per day) plus standard management of AA and AR for 30 days, while 28 received standard management only. All subjects supplemented with quercetin phospholipids showed a more significant decrease in daily symptoms and night symptoms, a better improvement in peak expiratory flow (PEF)/forced expiratory volume in the first second (FEV)_1_, and an improvement in PEF variability (expressed as a decrease of PEF variability) compared with subjects managed with SM only. In particular, subjects using the higher dose of supplementation had significantly better results (*p* < 0.05). Moreover, subjects supplemented with 500 mg of quercetin phospholipids used less rescue medication compared to the control group (3/20 subjects (15%) versus 8/28 (28.56%), *p* < 0.05).

An RDBPC study was conducted to investigate the effects of a 4-week oral intake of quercetin phospholipids on pollen-sensitized adult patients affected by AR. In total, 66 patients were randomized into two groups; one group of 33 received 200 mg of quercetin phospholipids, and the other group of 33 received a placebo. In the active group, the absolute values of the Japanese Rhino-conjunctivitis Quality of Life Questionnaire (JRQLQ) total score, Quality of Life (QOL) total score, sleep score, and physical score were significantly lower in the test food group than in the placebo group. The magnitude of change in sleep and physical score at 4 weeks was significant between the two groups (*p* = 0.000). Furthermore, the effects of quercetin were noticeable from the first week of treatment, where a significant decrease in the “ocular itching sensation” score was observed in the active group 1 week after the start of ingestion of the food supplement (*p* = 0.02) [72].

A third study evaluated the effects of quercetin phospholipids supplementation on recovery and performance in amateur triathlon athletes during a 14-day training regimen. Athletes in the supplementation group (250 mg twice daily) showed greater improvements in run completion time (−11.3% vs. −3.9%), subjective training value, and reduced post-training pain, cramps, recovery time, and oxidative stress compared to controls (*p* < 0.05). The findings suggest that quercetin phospholipids supplementation enhances training outcomes and recovery by reducing oxidative stress, highlighting its potential role in improving performance and recovery in endurance sports [75].

Another study assessed the antihistaminic effects of quercetin formulated in phospholipids on histamine-induced skin reactions and capillary permeability in healthy individuals. Participants consumed either 500 mg/day or 250 mg/day of quercetin phospholipids for 3 days, while controls received no supplementation. The supplementation significantly reduced wheal and redness areas, microcirculation flux, and skin thickness at histamine injection sites, with greater effects at the higher dose. Capillary filtration in the lower limbs was also significantly lower in supplemented subjects. The study demonstrated the dose-dependent antihistaminic and anti-edematous effects of quercetin phospholipids’ supplementation and highlighted its safety, suggesting potential applications in conditions linked to histamine release and oedema [76].

Regarding the anti-inflammatory, antioxidant, senolytic and immunomodulatory properties of quercetin [82], other human evidence has demonstrated the health benefits of a lecithin-based formulation. An RDBPC trial investigated the effects of daily supplementation with quercetin phospholipids over a 2-month period in 78 individuals experiencing chronic fatigue (CF) symptoms [83]. Among them, 40 participants received quercetin phospholipids (250 mg twice daily), while the remaining 38 participants were given a placebo. The findings highlighted significant improvements in fatigue, sleep quality, step count, and muscle performance in the group receiving quercetin phospholipids compared to the placebo group, indicating that quercetin phospholipids supplementation may effectively alleviate CF symptoms.

A recent retrospective case series explored the potential benefits of the lecithin formulation of quercetin in modulating chronic inflammation in fifty subjects with erosive hand osteoarthritis [84]. The study found that a daily supplementation of quercetin phospholipids at 500 mg for 30 days significantly reduced both the Numerical Rating Scale (NRS) score and the Functional Index for Hand Osteo-Arthritis (FIHOA). No side effects were reported, suggesting that the supplementation of this quercetin formulation was a safe and effective option for joint health and pain management as an additional natural support.

The good tolerability and safety profile for the use of the lecithin-based formulation of quercetin was confirmed based on the extensive documentation previously reported and supported by a large number of subjects supplemented with a dosage range from 250 mg to 1500 mg per day, up to a maximum of 3 months, without mild, moderate, or serious side effects.

The safety of this innovative food-grade delivery form of quercetin was further confirmed by assessing the potential interactions between quercetin phospholipids and various medications, including antiplatelet agents, anticoagulants, and diabetic therapy. The research evaluated the effects of quercetin phospholipids supplementation on bleeding time (BT) in patients taking antiplatelet drugs, International Normalized Ratio (INR) in patients on anticoagulants, and glycemia and glycated hemoglobin levels in diabetic patients. The results showed that quercetin supplementation did not significantly alter the antiplatelet activity, INR values, or metabolic control in diabetic patients, suggesting that quercetin phospholipids can be safely used alongside these common medications without causing adverse interactions [85].

Quercetin phospholipids also had proven health benefits when associated with other compounds. A recent study assessed the potential benefits of a combination of quercetin phospholipids and zinc (tablets containing 250 mg of Quercetin Phytosome™ and 5 mg of zinc) in alleviating symptoms of chronic allergic and non-allergic rhinitis [86]. A total of 36 subjects were enrolled and divided into three groups: mild chronic allergic rhinitis, vasomotor non-allergic rhinitis, and chronic drug-induced rhinitis. Each participant received two tablets of this supplement daily for 60 days. The study monitored symptoms such as nasal obstruction, rhinorrhea, and sneezing at baseline and after the supplementation period. The results showed that combination of quercetin phospholipids and zinc effectively alleviated symptoms, confirming the beneficial properties of quercetin, proposing its use as a support aid in nasal–sinus disorders, especially of allergic origin.

Based on the positive findings and proven safety in these studies, a new multicomponent product for children was formulated. This food supplement contains quercetin phospholipids (200 mg, of which 80 mg of quercetin), zinc (3.5 mg), and vitamin C (40 mg). The recommended dosage for children aged 3–6 is 200 mg per day, while for children over 6, it is 400 mg per day.

A preliminary study evaluated the effects of this new multicomponent food supplement alongside antihistamine therapy in children with seasonal allergic rhinitis (SAR). In total, 30 children (mean age 10.7 years) were divided into two groups: 15 received antihistamines alone, while 15 received antihistamines plus the supplement for two months. Symptom severity (Total Nasal Symptoms Score, TSS) decreased significantly in the supplement group (from 4.3 to 1.7, *p* < 0.05), whereas the antihistamine-only group showed a less marked improvement (from 4.2 to 2.9) and symptom relapse post-treatment. The supplement was well tolerated, suggesting potential benefits for SAR management [87].

Moreover, another potential application of quercetin may be the possible prevention of respiratory infections, mainly in allergic subjects. Namely, allergic subjects may be more prone to contract infections than healthy subjects [88]. This condition may depend on the type 2 polarization, diminishing type 1 immunity devoted to fighting infections, ICAM-1 overexpression on epithelial cells (ICAM-1 is the primary receptor for rhinoviruses), and chronic inflammation that promotes microbial rooting [89]. Moreover, respiratory viral infections are more common than bacterial ones [90]. Moreover, respiratory viral infections may favor a successive bacterial co-infection [91]. As a result, an additional antiviral activity may be envisaged as a particularly relevant property for quercetin in clinical practice. In this regard, there is evidence that quercetin may inhibit the replication of the hepatitis C virus [13]. Quercetin may also inhibit the replication of rhinoviruses and reduce the production of pro-inflammatory cytokines released after rhinovirus infection [92].

Furthermore, quercetin may prevent the cell entry of influenza A virus, including H1N1s and H3N2 types [12]. Finally, quercetin may inhibit the proteases essential for replication of SARS-Cov-2 [93]. This potential benefit was also confirmed by two pilot, randomized, controlled, and open-label clinical trials in early-stage SARS-CoV-2 infected people, in which quercetin phospholipids supplementation has been shown, in combination with standard care, to shorten the timing of molecular test conversion from positive to negative and, at the same time, to reduce the symptom severity and negative predictors of SARS-CoV-2 [94,95].

However, these preliminary experimental and clinical data require further evidence to confirm these outcomes.

## 9. Conclusions

Quercetin has emerged as a promising natural compound with significant potential in managing allergic diseases, primarily due to its anti-inflammatory, immunomodulatory, and antioxidant properties. The literature provides abundant in vitro evidence supporting its capacity to modulate Th2-driven inflammation, a hallmark of allergic disorders. Quercetin exerts its effects by targeting key inflammatory mediators while regulating immune cell activation and cytokine production. These mechanisms are complemented by their ability to counteract oxidative stress, a crucial factor in exacerbating inflammation in allergic conditions. These properties suggest that quercetin could serve as an effective antiallergic agent.

In vivo studies, particularly in animal models, provide strong evidence for quercetin’s efficacy. Models of AR in mice have consistently demonstrated its ability to alleviate symptoms such as nasal congestion, sneezing, and inflammation. These effects are mediated by reducing IgE levels, suppressing pro-inflammatory cytokines like IL-4 and IL-5, and restoring immune balance by promoting Th1/Th2 and Treg/Th17 homeostasis. Furthermore, quercetin has been shown to inhibit mast cell degranulation, decrease neuropeptide release, and modulate pathways like NF-κB, highlighting its broad spectrum of action in mitigating allergic responses. Similar findings have been reported in other allergic models, including conjunctivitis, asthma, atopic dermatitis, and food allergy, reinforcing the compound’s potential versatility.

However, bioavailability is still a drawback for wide use in clinical practice. Thus, the new lecithin-based formulation of quercetin, quercetin phospholipids, may offer a valuable advantage by assuring an ameliorated solubility and bioabsorption of quercetin and, consequently, optimal clinical outcomes.

On the other hand, despite these encouraging preclinical findings, human studies on quercetin remain limited. Most clinical research has focused on oral food supplements containing multiple nutraceuticals, making it challenging to isolate and evaluate the specific contribution of quercetin. Only three clinical trials have assessed quercetin as a standalone supplementation in allergic diseases. These studies reported positive outcomes, demonstrating reduced symptoms and improved quality of life. However, the small sample sizes and lack of robust trial designs limit the generalizability of these findings. Randomized controlled trials with larger cohorts and standardized protocols are needed to establish quercetin’s efficacy, optimal dosage, and safety profile in the clinical management of allergic diseases.

In conclusion, quercetin could be a potential tool for allergic conditions. Its ability to target multiple pathways involved in type 2 inflammation and oxidative stress makes it a compelling candidate for further exploration. However, translating these findings into clinical practice will require well-designed human studies to confirm its potential and guide its integration into clinical strategies. With more rigorous research, quercetin could complement existing treatments and improving patient outcomes with allergic diseases. In this regard, the Quercetin Phytosome formulation may represent a cornerstone in quercetin’s practical use.

## Figures and Tables

**Table 1:** Preclinical evidence on the mechanisms of action of quercetin.

Cell/Tissue/Species	Effect	Authors, Year [Reference]
***IMMUNOMODULATION ACTIVITY***		
PMACI-stimulated human KU812 cells	Inhibition of histamine release and cytokines	Li et al., 2016 [7]
Human keratinocytes HaCat cells AD-like dermatitis	Inhibition of Chemokine and cytokines	Hou et al., 2019 [8]
LAD2 Human Mast Cells	Inhibition of Mrgprx2-induced pseudo-allergic reaction	Ding et al., 2019 [9]
LAD2 Human Mast Cells	Reduce MC degranulation and inhibits Lyn kinase	Ding et al., 2020 [10]
***ANTIVIRAL ACTIVITY***		
Human Cytomegalovirus cell line	Inhibition of viral activity	Cotin et al., 2012 [11]
Influenza A virus strains	Inhibition of influenza A viral activity	Wu et al., 2016 [12]
Hepatitis C virus	Inhibition of hepatitis C viral activity	Bachmetov et al., 2012 [13]
SARS-CoV-2	Inhibition of SARS-CoV-2 viral activity	Kaul et al., 2021 [14]
***ANTIOXIDANT ACTIVITY***		
Co-culture of primary neutrophils	inhibited LPS-induced inflammatory	Wei et al., 2025 [15]
Mice platelets	Inhibition of platelet activation and Endoplasmic Reticulum-stress mediated autophagy	Manikanta et al., 2025 [16]
Review	Antioxidant activity	Xu et al., 2019 [17]
**ANTI-INFLAMMATORY ACTIVITY**		
Thymocytes and splenocytes	Inhibition of NF-κB and JAK/STAT signaling	Das et al., 2024 [18]
HUVEC cells	Inhibition of TNF-α	Chen et al., 2020 [19]
Allergic Asthma rat model	Reduction of oxidative stress	Rajizadeh et al., 2023 [20]

Abbreviations: PMACI: phorbol-12-myristate 13-acetate plus the calcium ionophore A23187; KU812: KU812 cell line; AD: atopic dermatitis; LAD: Laboratory of allergic disease; Mrgprx2: Mas-related G-protein coupled receptor member X2; SARS-CoV-2: Severe acute respiratory syndrome coronavirus 2; LPS: Lipopolysaccharide; NF-κB: nuclear factor kappa-light-chain-enhancer of activated B cells; JAK/STAT: Janus kinase/signal transducers and activators of transcription; HUVEC: Human Umbilical Vein Endothelial Cells; TNF: tumor necrosis factor.

**Table 2:** In vitro studies.

Type of Cell, and Intervention	Dosage	Effect	Authors, Year [Reference]
HNEpC stimulated with H_2_O_2_ treated with QUE	0.0, 0.1, 0.5, 1.0, 5.0, and 10 nM	QUE at concentrations greater than 1.0 nM induced significantly increased TRX levels in culture supernatants compared to those levels in the controls.	Edo et al., 2018 [47]
PMACI-stimulated human KU812 cells, incubated with isoQUE	12.5, 25, or 50 μg·mL^−1^	IsoQUE at 25 or 50 μg·mL^−1^ reduced the production of histamine and the pro-inflammatory cytokines (IL-6, IL-8, IL-1β, TNF-α) and suppressed MAPK and NF-κB.	Li et al. 2016 [7]
LAD2 human MC, incubated with QUE	0, 50, 100, 200, and 400 μM	QUE at greater concentration than 100 μM reduced Ca^2+^ fluxes and attenuated the release of β-hexosaminidase, histamine, MCP-1, and IL-8.	Ding et al., 2019 [9]
LAD2 human MC, incubated with QUE	0, 25, 50, 100 μM	Reduced MC degranulation and inhibited Lyn kinase.	Ding et al., 2020 [10]
HNEpC stimulated with IL-4 in the presence of QUE	0, 2, 4, 6, 8, 10 μM	QUE at a concentration of 4.0 μM suppressed the production of periostin in HNepC.	Irie et al., 2016 [48]
HNEpC stimulated with IL-4 in the presence of QUE	100 pM, 1–25–50–100 nM, 1 µM	QUE at a concentration of 1 nM suppressed the production of NO in HNepC.	Ebihara et al., 2018 [49]
Contracted mice trachea treated with QUE	n.r.	QUE (IC 49.3 µM) attenuated contraction in the airway smooth muscle.	Luo et al., 2018 [50]
HNEpC stimulated with TNF-α, treated with QUE	1.0, 2.5, 5.0 or 7.5 µM	QUE at 5.0 µM and higher dosages caused a significant increase in the ability ofHNEpCs to produce CC10 after TNF-α stimulation.	Otaki et al., 2023 [51]
T cell CD4^+^ stimulated with IL-4 and treated with QUE	1.0–10.0 µM	QUE at a concentration of 5.0 µM suppressed IL-5 and IL-13 production through the suppression of transcription factor activation and cytokine mRNA expression, but abrogated the inhibitory action of IL-4 on INF-γ production.	Tanaka et al., 2020 [52]
Human keratinocites HaCat cells AD-like dermatitis, treated with QUE	n.r.	QUE could inhibit pro-inflammatory chemokines and cytokines.	Hou et al., 2019 [8]
Human keratinocites HaCat cells AD-like dermatitis, treated with QUE	1.5 µM–25 µM	QUE at the concentration of 1.5 µM regulated expression of inflammatory mediators, supported the antioxidant defense system, promoted wound repair, and inhibited TSLP secretion.	Beken et al., 2020 [53]

Abbreviations. HNEpC: Human nasal epithelial cells; QUE: Quercetin; TRX: thioredoxin; PMACI: phorbol-12-myristate 13-acetate plus the calcium ionophore A23187; IL: Interleukin; TNF: tumor necrosis factor; MAPK: Mitogen-activated protein kinase; NF-κB: nuclear factor kappa-light-chain-enhancer of activated B cells; LAD: Laboratory of allergic disease; MC: mast cells; MCP-1: Monocyte chemoattractant protein-1; NO: nitric oxide; IFN: Interferon; CD: cluster of differentiation; AD: atopic dermatitis; TSLP: Thymic stromal lymphopoietin.

**Table 3:** In vivo studies: animal experiments.

Intervention	Concentration	Effect	Authors, Year [Reference]
QUE per os	0, 10, 15, 20, and 25 mg/kg	QUE at a higher dosage than 20 mg/kg decreased lipid peroxide levels and increased TRX levels in the nasal lavage fluids of the mice.	Edo et al., 2018 [47]
QUE i.v.	0, 1.0, 2.0, and 4.0 mg/mL	Reduce vasodilation and release of histamine and thickness of paw edema in dose-dependent manner.	Ding et al., 2019 [9]
QUE per os at low-medium-high dose for 7 days	1.0, 2.0, and 4.0 mg/kg	Inhibition of secretion of IgE, Il4, Tnf-α.	Ding et al., 2020 [10]
QUE per os at different dosages for 5 days	10, 20, 25, or 30 mg/kg	QUE at more than 25 mg/kg significantly increased CC10 levels in nasal lavage fluids	Otaki et al., 2023 [51]
Topical application of QUE	n.r	Improved AD lesions and reduced skin tissue level; of IFN-γ, CCL17, CCL22, TNF-α, IL-4, and IL-6.	Hou et al., 2019 [8]
QUE per os for 5 to 7 days	10–30 mg/kg/day for 2–7 days	QUE reduced symptoms in mice AR during nasal challenge and inhibited the increase in SP, CGRP, and NGF contents in nasal lavage fluids induced by nasal challenge.	Kashiwabara et al., 2016 [54]
QUE nasal administration	n.r.	Decrease symptoms and mucosal levels of IgE, IL-17, TNF-α, and IL-6/	Mu et al., 2024 [55]
IP QUE administration	80 mg/kg/die	Lower specific IgE for OVA and decreased expression of COX-2 and VIP in the quercetin and nasal mometasone group.	Sagit et al., 2017 [56]
IP QUE for 2 weeks	20–50 mg/kg/day for 13 days	QUE inhibited nasal symptoms of AR, promoted Th1/Th2 balance in the serum and NALF, inhibited inflammatory infltration, goblet cells, and eosinophils in nasal tissue and inhibited activation the NF-κB pathway.	Xia et al., 2023 [57]
Administratio of conjugated WP with QUE	n.r.	Conjugation of WP with quercetin reduced its allergenicity, even promoting Th1/Th2 and Treg/Th17 balance.	Ma et al., 2024 [58]
IP QUE administration	16 mg/kg/day	QUE was associated with lower epithelial, and subepithelial smooth muscle thickness, and goblet and mast cell numbers compared to untreated mice with allergic airway inflammation (*p* < 0.05). IL-4, IL-25, IL-33, and TSLP levels in BALF and OVA-specific IgE in serum were lower in quercetin-treated mice compared to the control group (*p* < 0.05).	Sozmen et al., 2016 [59]
IP QUE administration	50 mg/kg/day for 7 days	QUE reduced oxidative stress, TH_2_ citokynes levels, and the expression of GATA-3, α-SMA, IL-1β, TNFα, and TGF-β genes in lung tissue.	Rajizadeh et al., 2023 [20]

Abbreviations: AR: allergic rhinoconjunctivitis; QUE: quercetin; IgE: immunoglobulin E; IL: Interleukin; TNF: tumor necrosis factor; CC10: Clara cell 10-kD protein; IFN: interferon; CCL: chemokine ligand; IP: intraperitoneal; Th: T helper; NALF: nasal lavage fluid; NF-κB: nuclear factor kappa-light-chain-enhancer of activated B cells; WP: Walnut Protein; TSLP: Thymic stromal lymphopoietin; BALF: Broncho Alveolar Lavage Fluid; GATA-3: binding protein 3; α-SMA: α-smooth muscle actin; TGF: tumor growth factor.

**Table 4:** In vivo studies: multi-component nutraceuticals (containing quercetin among ingredients) studies on humans.

Type of Study	Number of Patients and Characteristics	Type of Intervention	Effects Described	Authors, Year [Reference]
RCDB	58 adult patients with AR (26 treated with Biminne (11 chinese herbs, among these *Ginkgo biloba*) and 32 received placebo)	Biminne (Rehmannia glutinosa 460 mg, Scutellaria baicalensis 460 mg, Polygonatum sibiricum 368 mg, Epimedium sagittatum 460 mg, Psoralea corylifolia 460 mg, Wu Wei Zi Schisandra chinensis 368 mg, Prunus mume 184 mg, Ledebouriella divaricata 460 mg, Angelica dahurica 368 mg, and Astragalus membranaceus 552 mg, *Ginkgo biloba*) five capsules twice a day for 12 weeks	Statistically significant improvement in symptoms score and VAS.	Hu et al., 2002 [60]
RCDB	16 adults with AR (8 treated with shallot capsule + cetirizine and 8 treated with placebo + cetirizine)	Cetirizine + *Allium ascalonicum* L. (*shallot*) 3 g capsule once a day for 4 weeks	Oral supplementation with *shallot* was safe and improve patients’ overall symptoms more than placebo, especially for itchy nose and eyes.	Arpornchayanon et al., 2022 [61]
OL	23 adults with AR	Perilla frutescens 80 mg (as dry extract), quercetin 150 mg, and vitamin D3 (200 IU) once a day + SM for 4 weeks	Reduction of AR symptoms: 70% for symptom scores and 73% in use of anti-allergic drugs.	Ariano, 2015 [62]
RCDB	146 children >6 y; <12 y with AR (70 treated with nutraceutical + SM vs. 66 treated with placebo + SM)	Perilla frutescens 80 mg (as dry extract), quercetin 150 mg, and vitamin D3 (200 IU) + SM for 4 weeks	Both groups signifcantly reduced TSS, without between-group difference. 24 children had total symptom score worsened: 8 in the AG and 16 in the placebo group, the difference between treatments being signifcant (*p* < 0.05).	Marseglia et al., 2019 [63]
OL	128 children >6 y; <12 y with AR (64 treated with nutraceutical and 64 received placebo)	Perilla frutescens 80 mg (as dry extract), quercetin 150 mg, and vitamin D3 (200 IU) for 4–12 weeks	Significant difference between groups as only 16 children in the AG had an AR exacerbation vs 27 children (42.2%) of OG (*p* = 0.039). Rescue medication utilization was significantly lower in the AG (9.6 + 9 days and 28.5 + 27.2 days, *p* = 0.018).	Marseglia et al., 2019 [64]
O	63 patients with AR, 32 treated with nutraceutical +SM and 31 received placebo + SM	Observation after treatment with Perilla frutescens 80 mg (as dry extract), quercetin 150 mg, and vitamin D3 (200 IU) + SM for 4 weeks	The median number of days of antihistamine therapy in the AG was 15 and 30 in the the CG (*p* =0.008).	Tosca et al., 2020 [65]
O	53 patients with AR, 28 treated with nutraceutical +SM and 25 received placebo + SM	Observation after treatment with Perilla frutescens 80 mg (as dry extract), quercetin 150 mg, and vitamin D3 (200 IU) + SM for 4–12 weeks	After 1 year of observation, the AG had significantly higher MEF50 than the CG (*p* = 0.009).	Leonardi et al., 2020 [66]
O	53 patients with AR, 32 treated with nutraceutical +SM and 31 received placebo + SM	Observation after treatment with Perilla frutescens 80 mg (as dry extract), quercetin 150 mg, and vitamin D3 (200 IU) + SM for 4–12 weeks	In the AG, the number of RI was lower compared to the CG (*p* = 0.01). In the AG, the number of antibiotic courses was lower compared to the CG (*p* = 0.002).	Zicari et al., 2020 [67]
RCDB	60 adult patients with AR (30 treated with *Ginko Biloba* + HA eyedrops vs. 30 with HA eyedrops)	*Ginko biloba* (quercetin, ginkgetin, kaempferol, isorhamnetin, procyani-din, prodelphinidin, Ginkgolids A, B, C, J, M, and bilobalides) + HA eyedrops for 4 weeks	All patients treated with GB-HA showed a significant improvement of subjective symptoms compared to HA patients.	Russo et al., 2009 [68]
POC study	12 adult patients affected by AR and/or bronchial obstruction disease	Nasal application of extract of *Artemisia abrotanum* L. at occurrence of AR symptoms	Significant nasal and ocular symptom relief was rapid and almost complete as evidenced by an improvement in the symptom score.	Remberg et al., 2004 [69]
RCDB	148 children with asthma in remission (74 treated with *A. membranaceus* + SM) vs. placebo + SM	Budesonide, terbutaline and *A. membranaceus* (0.25 mg for patients <20 kg and 0.5 mg for patients >20 kg) aerosol for 3 months	Improved lung function, reduction exacerbation, reduction of IgE, IL-17,IL-23 in treatment group.	Wang et al., 2019 [70]

Abbreviations: RCDB: randomized controlled double blind; AR: allergic rhinitis; VAS: Visual analogue scale; OL: open label; y: years; SM: standard management; TSS: Total Symptoms Score; AG: active-group; OG: observation group; O: observational; POC: proof-of-concept study; HA: hyaluronic acid solution; IgE: Immunoglobulin E; IL: interleukin.

**Table 5:** In vivo studies: single-component nutraceuticals (containing only quercetin) studies on humans.

Type of Study	Number of Patients and Characteristics	Type of Intervention	Effects Described	Authors, Year [Reference]
RCDB	66 adult patients with AR (33 received QUE phospholipids vs. 33 placebo)	200 mg of QUE phospholipids for 4 weeks	Significant improvement in subjective evaluation (influence on sleep) and objective evaluation (severity of allergic rhinitis and nasal discharge eosinophil count).	Yamada et al., 2022 [72]
Pilot randomized, six-sequence/three-period crossover clinical trial (3 × 3 × 3 crossover design)	12 health adults	Oral administration of 500 mg of QUE or 250 mg of QUE phospholipids or 500 mg of QUE phospholipids	Quercetin phospholipids demonstrated a better solubility and bioavailability than QUE.	Riva et al., 2019 [73]
Pilot study	58 adult patients with AA and AR (30 received nutraceutical + SM vs. 28 received placebo + SM)	250 or 500 mg/day of quercetin phospholipids + SM for 30 days	Quercetin phospholipids + SM showed superior results compared with SM alone in controlling, preventing, and reducing daily and night symptoms; in maintaining higher PEF; and in decreasing variability.	Cesarone et al., 2019 [74]
Pilot study	48 amatour healthy athletes (23 with QUE phospholipids and 25 did not) who underwent repeated triathlon sessions	500 of quercetin phospholipids for 2 weeks	In quercetin phospholipids the improvement of time to complete the run was greater (−11.3% vs. −3.9%; *p* < 0.05). Training was considered more valuable (*p* < 0.05). Post-run muscular pain, cramps, localized pain and the post-exercise recovery time were all considered better (*p* < 0.05). Oxidative stress was also reduced (*p* < 0.05).	Riva et al., 2018 [75]
Pilot study	57 healthy subjects (27 received quercetin phospholipids, 30 did not receive any treatment) received an injection of histamine, and wheal was measured	14 received 250 mg of quercetin phospholipids, 13 received 500 mg of quercetin phospholipids in the 3 previous days	The reaction to histamine was reduced in the groups supplemented with quercetin phospholipids. The higher dose was more effective. CF in supplemented subjects was also significantly lower after 3 days of quercetin phospholipids consumption, in comparison with controls.	Belcaro et al., 2020 [76]

Abbreviations: AA: allergic asthma; AR: allergic rhinitis; QUE: quercetin; SM: standard management; PEF: peak expiratory flow; RCDB: randomized controlled double blind; CF: capillary filtration.
